# Enhanced fermentative performance under stresses of multiple lignocellulose-derived inhibitors by overexpression of a typical 2-Cys peroxiredoxin from *Kluyveromyces marxianus*

**DOI:** 10.1186/s13068-017-0766-4

**Published:** 2017-03-28

**Authors:** Jiaoqi Gao, Hualiang Feng, Wenjie Yuan, Yimin Li, Shengbo Hou, Shijun Zhong, Fengwu Bai

**Affiliations:** 1 School of Life Science and Biotechnology, Dalian University of Technology, Dalian, 116024 China; 2 State Key Laboratory of Microbial Metabolism, Shanghai Jiaotong University, Shanghai, 200240 China

**Keywords:** Lignocellulose, Inhibitors, Tolerance, Peroxiredoxin, Ethanol fermentation, *Kluyveromyces marxianus*

## Abstract

**Background:**

Bioethanol from lignocellulosic materials is of great significance to the production of renewable fuels due to its wide sources. However, multiple inhibitors generated from pretreatments represent great challenges for its industrial-scale fermentation. Despite the complex toxicity mechanisms, lignocellulose-derived inhibitors have been reported to be related to the levels of intracellular reactive oxygen species (ROS), which makes oxidoreductase a potential target for the enhancement of the tolerance of yeasts to these inhibitors.

**Results:**

A typical 2-Cys peroxiredoxin from *Kluyveromyces marxianus* Y179 (*KmTPX1*) was identified, and its overexpression was achieved in *Saccharomyces cerevisiae* 280. Strain TPX1 with overexpressed *KmTPX1* gene showed an enhanced tolerance to oxidative stresses. Serial dilution assay indicated that *KmTPX1* gene contributed to a better cellular growth behavior, when the cells were exposed to multiple lignocellulose-derived inhibitors, such as formic acid, acetic acid, furfural, ethanol, and salt. In particular, *KmTPX1* expression also possessed enhanced tolerance to a mixture of formic acid, acetic acid, and furfural (FAF) with a shorter lag period. The maximum glucose consumption rate and ethanol generation rate in *KmTPX1*-expressing strain were significantly improved, compared with the control. The mechanism of improved tolerance to FAF depends on the lower level of intracellular ROS for cell survival under stress.

**Conclusion:**

A new functional gene *KmTPX1* from *K. marxianus* is firstly associated with the enhanced tolerance to multiple lignocellulose-derived inhibitors in *S. cerevisiae*. We provided a possible detoxification mechanism of the *KmTPX1* for further theoretical research; meanwhile, we provided a powerful potential for application of the *KmTPX1* overexpressing strain in ethanol production from lignocellulosic materials.

**Electronic supplementary material:**

The online version of this article (doi:10.1186/s13068-017-0766-4) contains supplementary material, which is available to authorized users.

## Background

Fuel ethanol has been the earliest and most mature biofuel product so far and widely considered as one of the most promising biomass energies. Bioethanol from lignocellulosic materials represents the most promising renewable fuel due to its wide range of sources. However, the complex structure of lignocellulose requires a pretreatment step to produce monosaccharides for *Saccharomyces cerevisiae*. Current efficient pretreatment of lignocellulose by steam explosion, acids, or alkali may generate inhibitors to restrain the growth of yeasts in the fermentation, which represents a great challenge for the scale-up of ethanol production from lignocellulosic materials [1].

Lignocellulose-derived inhibitors in hydrolysates include weak acids (formic acid and acetic acid), furan derivatives (furfural and 5-hydroxymethyl furfural), and phenolic compounds (phenol and *O*-methoxyphenol) [1]. Toxic mechanisms of inhibitors in lignocellulosic hydrolysates are supposed to be extremely complicated. The inhibitors may be removed by detoxification of lignocellulosic hydrolysates [2], which should not be regarded as the preferred choice considering loss of sugars and increased costs. Therefore, exploring the toxicity of distinctive inhibitors to cells and its mechanism, and developing excellent strains with enhanced tolerance are becoming a more critical component of ethanol production from lignocellulosic materials. However, mechanisms of toxicities of these inhibitors in yeasts are very complex and greatly variable depending on strains [3].

Inhibitors like acetic acid, furfural, and phenol have been reported to be related to the redox state inside cells, inducing reactive oxygen species (ROS) generation [4–6]. Acetic acid generally affects cell metabolism and stabilities of proteins by a drop in intracellular pH and potential, leading to a net negative effect on yeasts’ cell growth and proliferation [7]. Acetic acid diffusing across plasma membrane damages cells by accumulating ROS [4], and therefore the expression of some oxidoreductases like mitochondrial cytochrome C oxidase chaperone gene (encoded by *COX20*) may improve tolerance to weak acids, especially acetic acid in *S. cerevisiae* [8]. Moreover, an enhancement of intracellular proline concentration by the addition of proline or overexpression of a proline synthesis-related gene (*PRO1*) led to an obvious increase in tolerance to both acetic acid and furfural [9]; unlike acetic acid, the toxicity of furfural results from the inhibition of glycolytic and fermentative enzymes essential to central metabolic pathways, which reduces cell growth rates eventually [6]. Interestingly, furfural also induces the accumulation of ROS inside cells by lowering activities of intracellular oxidoreductases [10]. Consequently, some oxidoreductases, such as alcohol dehydrogenase *ADH1* [11] and *ADH6* [12], 3-methylglyoxal reductase *GRE2* [13], aldehyde reductase *ARI1* [14], and xylose reductase *XYL1* [15], help yeast cells convert furfural into the less toxic alcohols and then increase tolerance to furfural; phenolic compounds alter the permeability of biological membranes and caused irreversible damages to the cells. Phenolic compounds are supposed to be even more toxic than furfural by generating ROS like peroxides and superoxides inside the cells [6]. It has been indicated that oxidoreductases could be quite effective in enhancing the tolerance of yeast cells to phenolic compounds in lignocellulosic hydrolysates [16]. But beyond that, ionic liquids and hydrogen peroxide have become a new kind of inhibitors as the pretreatment technologies evolve. Hence, oxidoreductases have shown a perfect application in the enhancement of tolerance to multiple inhibitors from pretreatment of lignocellulose.

High-throughput sequencing is a powerful tool to gain insight into new genes and their new functions [16–19]. Our previous study on the transcriptional analysis of the non-conventional yeast *Kluyveromyces marxianus*, which possesses the advantages of high-temperature resistance, rapid growth rate, and diversity of substrates, identified lots of differentially expressed genes (DEGs) [20]. A greatly up-regulated gene under aerobic conditions (ORP controlled at −130 mV) from *K. marxianus* Y179 has attracted our attention. According to the sequence alignment, this gene (*KmTPX1*) belongs to a large and highly conserved peroxiredoxin family. *KmTPX1* is homologous to one of the five peroxiredoxins (Prxs) in *S. cerevisiae* (*TSA1*/*TPX1*) [21], which is the most abundant Prxs in cells [22]. Tsa1p in *S. cerevisiae* has been reported to participate in oxidation–reduction reactions to remove excess ROS like peroxides [23], and the antioxidant role of Tsa1p in regulating the concentrations of intracellular peroxides protects cells from DNA damage and cell death [24, 25]. However, so far, no reports have combined Tsa1p in *S. cerevisiae* with tolerance to lignocellulose-derived inhibitors, and the homologous protein in *K. marxianus* (encoded by *KmTPX1*) has never been studied.

Therefore, in this study, *KmTPX1* gene was cloned and then overexpressed in *S. cerevisiae* to analyze its potential functions in yeast cells. Afterwards, tolerance of the recombinant *S. cerevisiae* to multiple inhibitors or stressors in lignocellulosic hydrolysates was evaluated. The enhanced tolerance to formic acid, acetic acid, furfural, and salt makes great difference to ethanol production from lignocellulosic materials and provides more theoretical references for its industrial scale-up in the future.

## Results and discussion

### Identification of a hypothetical typical 2-Cys peroxiredoxin from *K. marxianus*

**Fig. 1:**
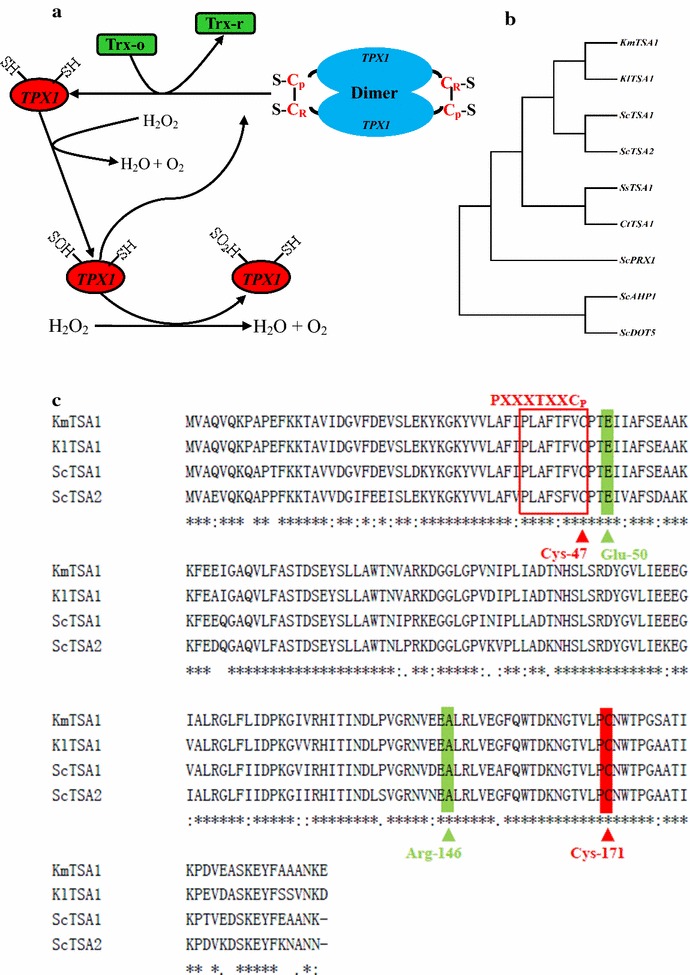
Typical 2-Cys peroxiredoxin in common yeasts. **a** A typical catalytic cycle of *TPX1* protein as a dimer structure. **b** Evolutionary tree of some typical 2-Cys Prxs from Y179 and their allied species using *MEGA 4* software [41]. **c** Alignment of amino acid sequences of typical 2-Cys Prxs from Y179, *K. lactis*, and *S. cerevisiae* indicates some active domains and sites

Sequence analysis of *KmTPX1* gene from *K. marxianus* Y179 was conducted in this study. *KmTPX1* can be classified into the typical 2-Cys Prx family, and the two conserved cysteine residues in *KmTPX1*, serving as the site of oxidation by peroxides, are named as “Peroxidatic” Cys (C_P_) and “Resolving” Cys (C_R_). To perform its functions in oxidation–reduction reactions, this typical 2-Cys protein needs to exist in dimer model, which indicates an inter-subunit disulfide formation between the C_P_-SOH and the C_R_-SH in the other subunit [23]. A specific catalytic cycle of *TPX1* protein is illustrated in Fig. 1a.

Furthermore, the construction of evolutionary tree by several typical 2-Cys Prxs from *S. cerevisiae*, *Kluyveromyces lactis*, *Scheffersomyces stipites*, and *Candida tropicalis* indicates that the evolutionary distance between *KmTPX1* and Prxs from *K. lactis* is the shortest, followed by Tsa1p from *S. cerevisiae*. The other four Prxs from *S. cerevisiae* have the lowest homologies with *KmTPX1*, which makes us even more certain that *KmTPX1* should be identified as a hypothetical typical 2-Cys Prx (Fig. 1b).

Simultaneously, a simple function annotation was conducted using amino acid sequence from *K. marxianus*,Y179. Firstly, we determined the accurate positions of two cysteine residues in *KmTPX1*, which were found at the sites of 47 and 171, respectively. Besides, an active domain, PXXXTXXC_p_, has been reported to exist in all Prxs [26], and this domain lies in amino acids between the 40th and 47th sites in *KmTPX1*. Meanwhile, the 146th Arg, another active site, is supposed to be close to the above active domain in 3D structure while it is far away from C_p_ in 2D sequence [26, 27]. Finally, Tairum et al. [27] constructed the three-dimensional structure model of Tsa1 protein, which showed that the 50th Glu and the 46th Arg are two essential sites for disulfide formation. These two functional sites in *KmTPX1* were also found to be highly conserved from the alignment results (Fig. 1c).

### Enhanced tolerance of TPX1 strain to oxidative stresses

To validate the possible functions of *KmTPX1*, we constructed the overexpression vector containing the gene *KmTPX1*, and the overexpression was achieved in *S. cerevisiae* 280 after verification by restriction enzyme digestion. Meanwhile, real-time quantitative PCR was performed to test the levels of expression. Expression of *KmTPX1* gene in strain TPX1 can be detected, compared with the control strain 423 containing only empty vector (an additional file shows this in more detail [see Additional file 1]).

**Fig. 2:**
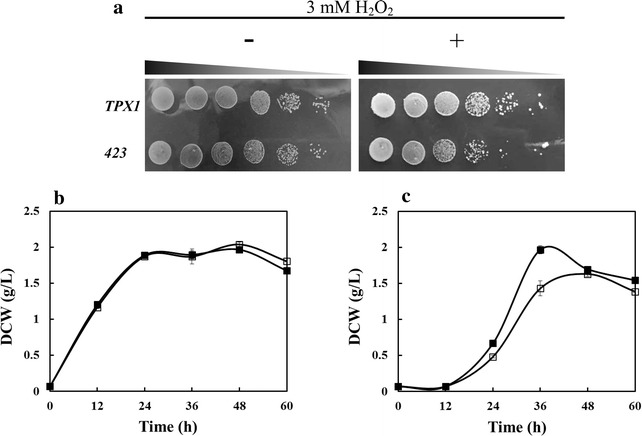
Effects of *KmTPX1* overexpression on the growth behavior under oxidative stress. **a** Serial dilution assay of strains TPX1 and 423 in the medium without or with 3 mM of H_2_O_2_. **b** Growth behavior of strains TPX1 (*filled square*) and 423 (*empty square*) in liquid fermentation medium containing 0.2 mM of H_2_O_2_. **c** Growth behavior of strains TPX1 (*filled square*) and 423 (*empty square*) in liquid fermentation medium containing 3 mM of H_2_O_2_

Prxs have been reported to play an important role in regulating intracellular redox state by the participation of oxidation–reduction reactions [23, 28]. Therefore, *KmTPX1* is supposed to be related to the removal of excess peroxides inside the cells for a much better chance of survival at high concentrations of ROS. On the one hand, serial dilution assay was conducted on SC-His agar plates with or without H_2_O_2_. As shown in Fig. 2a, no significant differences in growth were detected between TPX1 and 423 without H_2_O_2_. However, when 3 mM of H_2_O_2_ was added to the plates, TPX1 achieved a much better growth status than the strain 423, which indicated that the gene *KmTPX1* might increase the tolerance of *S. cerevisiae* to H_2_O_2_. Similarly, a kind of 2-Cys Prxs from *Oryza sativa* [29] and the thioredoxin from *Endocarpon pusillum* [30] both have been proved to enhance the tolerance of *S. cerevisiae* to peroxides, including H_2_O_2_ and 2-methyl-1,4-naphthoquinone (MD).

On the other hand, we carried out liquid fermentation experiments at different concentrations of H_2_O_2_ to further confirm our findings (Fig. 2b). There were no obvious differences in growth between two strains under low concentration (0.2 mM). An elevated concentration of 3 mM could inhibit the growth of the control strain 423 significantly, while strain TPX1 had been less affected, which was in line with the results reported by Kim et al. [29]. Consequently, an enhanced tolerance of *S. cerevisiae* 280 with a normally functioning *KmTPX1* gene provided a good foundation for further analysis and validation of other possible functions.

### Responses to multiple lignocellulose-derived inhibitors in *KmTPX1*-overexpressing yeast

Lignocellulose-derived inhibitors, such as acetic acid, furfural, and phenol, induce the generation of intracellular ROS that is closely related to the redox state in cells. Hence, we further explored the potential applications of gene *KmTPX1* in an enhanced tolerance of yeasts to the mixed inhibitors in the lignocellulosic hydrolysates. As a result, weak acids (formic acid and acetic acid), phenolic compounds (phenol and *O*-methoxyphenol), and furan derivative (furfural and 5-HMF), as the representative inhibitors in hydrolysates, were selected to test their effects on *S. cerevisiae* overexpressing *KmTPX1*.

**Fig. 3:**
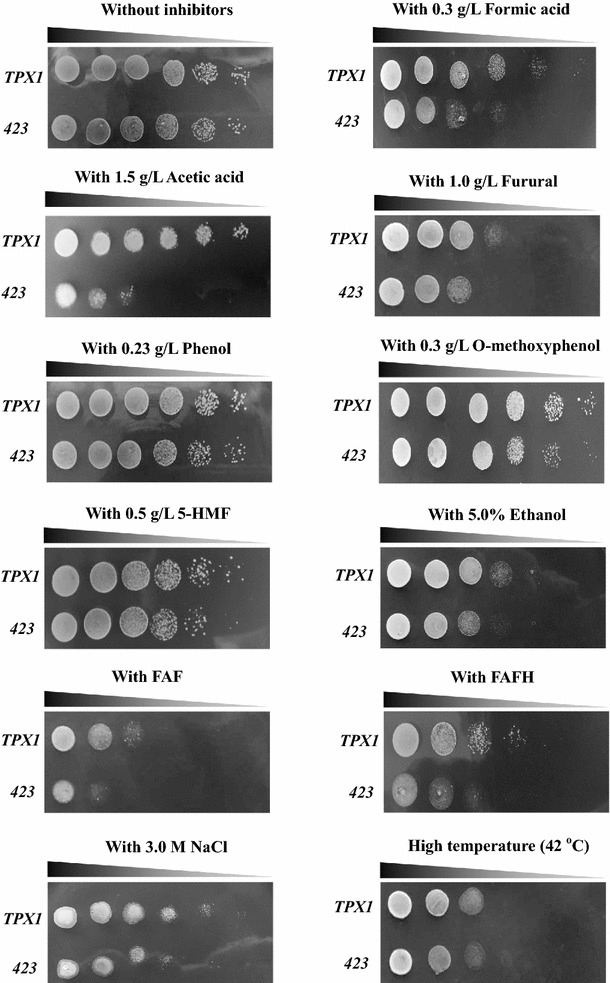
Stress response of *KmTPX1* overexpression to the presence of multiple lignocellulose-derived inhibitors by serial dilution assay. Cells with 10 g/L of DCW in log phase were serially diluted to 10^−5^ and then spotted onto SC-His plates containing various inhibitors. Cells were cultivated at 30 or 42 °C for 3 days and then photographed

As shown in Fig. 3, gene *KmTPX1* played a positive role in the enhanced tolerance to weak acids and furfural. Overexpression of *KmTPX1* would not affect the cell growth (Fig. 2a), while the growth of control strain 423 had been greatly repressed on plates with 0.3 g/L of formic acid, 1.5 g/L of acetic acid, or 1.0 g/L of furfural, which was 1–2 gradients less than the strain TPX1 in the serial dilution assay. These results have been supported by a variety of similar oxidoreductases that might be contributed to the increased tolerance of *S. cerevisiae* to the inhibitors [8–15, 29]. However, despite sharing a similar mechanism of toxicity with furfural, the tolerance of TPX1 strain to 5-HMF was only slightly improved compared with the control strain.

Considering the possibilities of applications in industrial-scale ethanol production from lignocellulosic materials, the enhanced tolerance to a single inhibitor makes little sense. So we further evaluated the abilities of *KmTPX1* to tolerate a mixture of lignocellulose-derived inhibitors. An improved tolerance of strain TPX1 to mixed FAF inhibitors was observed. Interestingly, a much more obvious difference in cell growth was detected when the two strains were exposed to mixed FAFH inhibitors, which might be attributed to the comprehensive stress compared with the FAF inhibitors. A similar publication reported that the addition of proline or overexpression of a proline synthesis-related gene (encoded by *PRO1*) might also achieve enhanced tolerance to a mixture of furfural, acetic acid, and phenol [9]. In this case, a generally elevated tolerance of yeasts to the mixed lignocellulose-derived inhibitors due to some key genes makes a major breakthrough in the biofuel industry.

Besides, our tests on phenol and *O*-methoxyphenol indicated that *KmTPX1* did not work for these two inhibitors despite marginally increased tolerance to *O*-methoxyphenol. Except for lignocellulose-derived inhibitors, phenol and *O*-methoxyphenol are also two typical environmental pollutants, which might make a great difference if we achieved both enhanced tolerance and degradation. The elevated effect of *KmTPX1* on these two inhibitors was not obvious, which can be attributed to two main reasons: on the one hand, test conditions were not quite suitable; on the other hand, some doubts remain on whether these two inhibitors will generate ROS.

In particular, we also evaluated the tolerance of strain TPX1 to salt, ethanol, and high temperature from a global perspective of ethanol production. An enhanced tolerance of strain TPX1 to high concentration of salt was just as expected, which showed similar results with some related Prxs [29, 30]. This finding is of great significance to reducing the costs for scale-up of ethanol production. Surprisingly, *KmTPX1* played a positive role in growth at high concentrations of ethanol. No previous results showed that ethanol had possible relations with intracellular redox states, so oxidoreductases might also be applied in increasing tolerance to ethanol. Therefore, this special result makes great sense in the field of ethanol fermentation. Finally, no significant effect of *KmTPX1* in cell growth at high temperature was observed in spite of the relations between high temperature and intracellular ROS [31]. In contrast, another Prx family protein, thioredoxin from *Endocarpon pusillum*, showed a positive role in increasing yeast tolerance to high temperature [30].

The principal advantage of *KmTPX1* gene tends to increase the tolerance to mixed lignocellulose-derived inhibitors. Although some other oxidoreductases have been proved to promote cell growth when exposed to a single inhibitor, a comprehensively enhanced tolerance to multiple stressors may provide more references and potential applications for ethanol production from lignocellulosic materials.

### Effect of initial pH on acetic acid tolerance in transgenic yeast cells

*KmTPX1* gene has shown a distinct effect on increasing tolerance of *S. cerevisiae* to multiple lignocellulose-derived inhibitors. In our experiments, a lack of adequate nutrition in SC medium led to an obvious reduction in tolerance to acetic acid. Concentrations of acetic acid in lignocellulosic hydrolysates have been reported to achieve up to 5 g/L [32], so the relatively low concentration of acetic acid in FAF affects its possibility of application in industrial-scale production. Ionization of some inhibitors under certain pH values may change the degree of toxicity of the compounds. Therefore, the initial pH of the medium was adjusted to improve cell growth as much as possible under the current conditions [33].

**Fig. 4:**
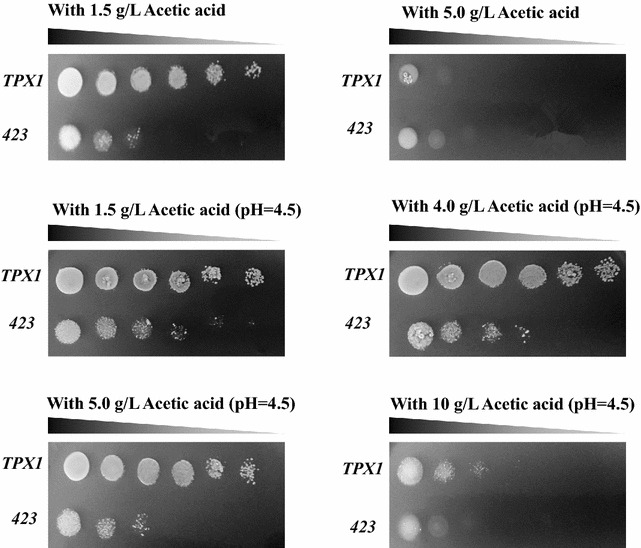
Effects of initial pH on the tolerance of yeast cells to acetic acid. Serial dilution assay was performed on SC-His plates containing different concentrations of acetic acid. To investigate the effects of pH, the initial pH in plates was adjusted to 4.5 with 3 N NaOH when required

Without adjusting the initial pH after the addition of inhibitors, overexpressing strain TPX1 could grow well in the medium with 1.5 g/L of acetic acid compared with the serious repression of control strain. Both strains stopped growth as the acetic acid amount was increased to 5 g/L; when the initial pH was adjusted to around 4.5 after the addition of inhibitors, an enhanced tolerance to acetic acid was detected. TPX1 was tolerant to 5 g/L of acetic acid. Under these conditions, both strains were not still able to survive in more than 10 g/L of acetic acid (Fig. 4). Even so, strain TPX1 that could tolerate 5 g/L of acetic acid in SC medium, which is supposed to be much higher in normal media, has already satisfied the requirement of industrial-scale ethanol production from lignocellulosic materials. Moreover, a mitochondrial cytochrome C oxidase chaperone gene (encoded by *COX20*) has also been reported to contribute to a perfect survival with the acetic acid concentration up to 75 mM (about 3.5 g/L) [4]. Thus, it can be seen that oxidoreductases have broad applications in increasing tolerance of *S. cerevisiae* to acetic acid, which may be further strengthened by adjusting the initial pH values.

### Batch fermentation by *KmTPX1*-overexpressing yeast with FAF

**Fig. 5:**
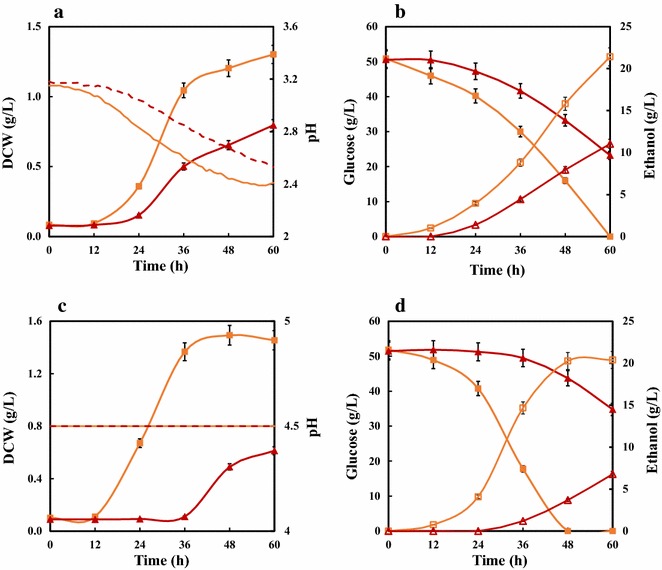
Batch ethanol fermentation by *KmPX1*-expressing *S. cerevisiae* under FAF stress. **a**, **b** Fermentative profiles of two strains within 60 h without controlling pH, or controlling pH at 4.5, respectively. **c**, **d** Fermentative profiles of two strains within 60 h when controlling pH at 4.5. Cells were pre-cultured in SC-His medium containing 1 mM H_2_O_2_ for 16–18 h. 5% of seed culture was inoculated into a 3-L bioreactor with a 1 L working volume. Data are given as mean ± SD, n = 2

To test the potential performance of ethanol fermentation using inhibitors in *S. cerevisiae* with *KmTPX1* gene, we conducted batch ethanol fermentation in both flasks (an additional file shows this in more detail [see Additional file 2, Additional file 3]) and bioreactors (Fig. 5; Table 1) containing 50 g/L of glucose and FAF inhibitors.

**Table 1:** Fermentative performance under FAF inhibitors in 3-L bioreactors

	TPX1	423	TPX1-pH^a^	423-pH^a^
Lag phase, h	0	24	0	36
Biomass, g/L	1.30 ± 0.06	0.80 ± 0.05	1.49 ± 0.12	0.61 ± 0.01
Initial glucose, g/L	51.84 ± 2.60	51.45 ± 1.89	50.86 ± 1.01	50.56 ± 2.27
Residual glucose, g/L	0	23.22 ± 1.24	0	34.78 ± 1.91
Ethanol, g/L	21.41 ± 0.83	11.03 ± 0.51	20.38 ± 1.02	8.50 ± 1.33
Glucose consumption rate, g/L/h	0.86 ± 0.04	0.47 ± 0.08	1.06 ± 0.02	0.33 ± 0.02
Productivity, g/L	0.36 ± 0.01	0.18 ± 0.01	0.42 ± 0.02	0.18 ± 0.03

^a^pH in the media with an elevated concentration of acetic acid was controlled at 4.5

As shown in Fig. 5 and Additional file 2: Figure S2, the overexpression of *KmTPX1* gene in cells helped accelerate the process of ethanol fermentation under inhibitors and greatly reduce the lag phase in both flasks and bioreactors, which showed a similar trend. Considering the experiments in the 3-L bioreactors as an example (Fig. 5; Table 1), fermentative performance was greatly enhanced whether controlling the pH or not. Without adjusting the initial pH after the addition of inhibitors (Fig. 5a, b), the lag phase was slashed by almost 24 h compared with that in the control strain. Glucose consumption rate and ethanol productivity of TPX1 were achieved up to 0.86 and 0.36 g/L/h, respectively, both of which were double those of the control strain. Particularly, a lower residual glucose concentration for strain TPX1 was observed because of an accelerated fermentative process (Table 1). This may make great differences to industrial-scale ethanol production.

In addition, controlling pH at 4.5 greatly improved both the fermentative performance and the tolerance to acetic acid in TPX1 strain. As mentioned above, the poor nutrition in SC media led to the decrease of acetic acid concentration in FAF inhibitors. Fortunately, adjusting the initial pH in serial dilution assay helped solve this problem. Therefore, fermentations were conducted in bioreactors to control the pH at around 4.5 in media throughout the process. As shown in Fig. 5c, d and Table 1, TPX1 strain could tolerate the FAF inhibitors containing an elevated concentration of acetic acid without an obvious lag phase. The glucose consumption and ethanol production achieved a reasonable result within 48 h; in contrast, the control strain hardly grew under the same conditions, which was mainly manifested in the extremely low consumption rate of glucose (less than 10 g/L within 48 h).

Consequently, *KmTPX1* gene overexpressed in *S. cerevisiae* increased the tolerance to lignocellulose-derived inhibitors, thus shortening the lag period and accelerating the fermentation process. Besides, a shorter lag period is supposed to be the general characterization for strains with an enhanced tolerance [9, 29]. We would see a better fermentative performance in strain TPX1, as was described previously by Kim et al. [29], if higher concentrations of initial glucose and final ethanol were adopted.

### Increasing stress tolerance related to intracellular ROS levels

Despite the complicated mechanisms that lignocellulose-derived inhibitors are toxic to cells, they may induce cells to generate intracellular ROS, either FAF or phenol [4–6]. Intracellular ROS damages cells by altering the growth and metabolism characteristics. Therefore, oxidoreductases help remove the excess ROS, maintaining it at a normal level, to achieve an elevated tolerance and reduce damages to cells. Intracellular ROS in cells with an increased tolerance has been reported to decrease under inhibitors, no matter what strategies were adopted [9, 29, 34].

**Fig. 6:**
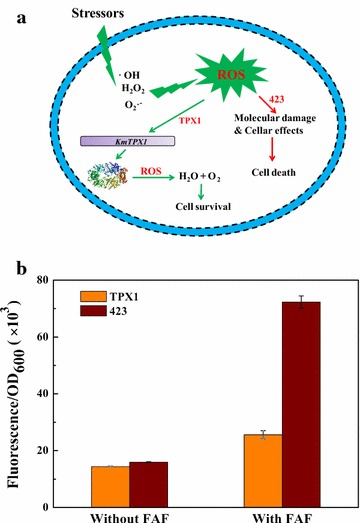
Regular levels of intracellular ROS contribute to cell survival. **a** A hypothesis on the mechanism of an enhanced tolerance to multiple inhibitors in TPX1 cells. **b** Detection of intracellular ROS with or without FAF inhibitors by DCFH-DA

According to our results and existing theories, the possible mechanism of *KmTPX1* gene is inferred to be related to the levels of intracellular ROS. When the cells were exposed to FAF inhibitors, ROS like ∙OH, H_2_O_2_, and O_2_∙^−^ might be generated. Hence, on the one hand, these ROS caused molecular damages and cellar effects to the normal cells, which eventually led to cell death [16]. However, on the other hand, overexpressed cells with *KmTPX1* gene achieved functional dimer proteins after transcription and translation under peroxides [23]. The activated dimers removed excess ROS inside the cells to maintain normal cell metabolism and to ensure a high rate of cell viabilities (Fig. 6a).

Fortunately, the measurement of intracellular ROS provided strong supports for our inference. Without FAF inhibitors, the level of intracellular ROS in TPX1 was slightly lower than that in 423, which was proved to have no differences. Rather, with inhibitors of FAF, the level of intracellular ROS in control strain increased dramatically, achieving up to 3 times more than that in strain TPX1, and a lower amount of ROS was an important guarantee of growth, metabolism, and multiplication of yeasts (Fig. 6b). Above all, our results and some previous reports all confirmed that oxidoreductases maintained a normal level of oxidoreductases, which eventually manifested as an apparently enhanced tolerance of *S. cerevisiae* to multiple lignocellulose-derived inhibitors [9, 29, 34].

## Conclusion

Ethanol production from lignocellulosic materials has been considered as one of the most promising substitutions for fossil fuels. In this study, through a comprehensive transcriptional analysis, we identified a typical 2-Cys peroxiredoxin from *K. marxianus* Y179 (*KmTPX1*). Meanwhile, the overexpression of *KmTPX1* gene has been proved to regulate the levels of intracellular ROS, which correspondingly increased the tolerance of *S. cerevisiae* to both oxidative stress and multiple lignocellulose-derived inhibitors. Particularly, a reasonable fermentation profile of the overexpressing strain TPX1 was achieved in the presence of FAF inhibitors. These findings lay a good foundation for further research on industrial-scale ethanol production from lignocellulosic materials.

## Methods

### Strains, media, and growth conditions

All strains used in this study are listed in Table 2. Yeast cells were grown at 30 °C in YPD medium (2% glucose, 1% yeast extract, and 2% peptone) or in synthetic complete medium (SC) containing 2% glucose and 0.67% yeast nitrogen base, supplemented with the appropriate amino acids. *Escherichia Coli* DH5α was cultivated at 37 °C in LB medium (0.5% yeast extract, 1% peptone, and 1% sodium chloride).

**Table 2:** Strains, plasmids, and primers used in this study

Strains	Genotype	Source
Y179	*K. marxianus*, wild-type	Lab stock
280	*S. cerevisiae*, *MAT* **a,** *his3*-*1, leu2*-*0, met 15*-*0, ura3*	Lab stock
DH5α	*E. coli*, for genetic manipulation	Lab stock
TPX1	*S. cerevisiae* overexpressing *KmTPX1* gene, transformed from 280	This study
423	*S. cerevisiae* with pRS423 plasmid, transformed from 280	This study

Plasmids	Characteristic	Source and reference
pRS423	Yeast episomal vector with *HIS3* marker	[37]
p423TPX	*KmTPX1* in pRS423	This study
Primers		
KmTPX1-F	5′-CTT*gagctc*AATGTCTCGTCTCGTCTCGT-3′	
KmTPX1-R	5′-TCC*ccgcgg*GGCTAAGCCAATAACTTATT-3′	
RT-TPX1-F	5′-CTCAAGTTTTGTTCGCTTCCAC-3′	
RT-TPX2-R	5′-AAGTCGTTGATGGTGATGTGTCT-3′	
ACT1-F	5′-ACGTTGTTCCAATCTACGCC-3′	
ACT1-R	5′-CTTGTTCGAAGTCCAAAGCG-3′	

### Plasmid construction and transformation

To construct an overexpression vector with the *KmTPX1* gene, plasmid pRS423 [35] was used for DNA manipulation and cloning. DNA manipulation was performed by standard procedures [36]. *KmTPX1* gene fragment was amplified from genome of *K. marxianus* Y179 with primers TPX1-F and TPX1-R. The obtained fragment with a coding region and a native promoter of *KmTPX1* gene was inserted into pRS423 to generate pRS423–TPX1 after digestion with *Sac*I and *Sac*II (Table 2; Additional file 1: Figure S1a). The constructs were verified by both digestion of restriction enzyme and sequencing (Additional file 1: Figure S1b).

Transformation of *E. coli* cells was performed using the CaCl_2_ method [37]. For screening the transformants, ampicillin (100 μg/mL) was added into LB selective plates. Yeast transformation was conducted using the LiAc/PEG method described by Gietz et al. [38], and the transformants were screened by SC medium without histidine (SC-His). Transformants with the recombinant plasmid of pRS423–TPX1 were marked as TPX1, and strains with the plasmid pRS423 were set as control (423) in this study. All transformants were verified by PCR.

### Stress tolerance assay

To test the functions of *KmTPX1* gene, serial dilution assay was performed using strain TPX1, and strain 423 was used as the control. Yeast cells were cultivated in SC-His medium with 1 mM H_2_O_2_ for 16–18 h at 30 °C and 150 rpm and then collected by centrifugation at 3000×*g* for 5 min. The collected cells (final cell density 10 g/L of DCW) were serially diluted with distilled water, after which 10 μL of the diluted cells was loaded onto SC-His agar plates containing a single or mixed lignocellulose-derived inhibitors. The inhibitors applied in this section included 3 mM H_2_O_2_, 0.3 g/L formic acid, 1.5 g/L acetic acid, 1.0 g/L furfural, 0.23 g/L phenol, 0.3 g/L *O*-methoxyphenol, 0.5 g/L 5-HMF, 5% (v/v) ethanol, 3 M NaCl, FAF mixture (0.3 g/L formic acid, 1.5 g/L acetic acid and 0.6 g/L furfural), and FAFH mixture (0.2 g/L formic acid, 0.8 g/L acetic acid, 0.3 g/L furfural, and 0.3 g/L 5-HMF). All plates were incubated for 2–3 days at 30 °C and then photographed. For high-temperature test, plates were incubated at 42 °C.

Fermentation experiments with H_2_O_2_ were also conducted to further verify the potential function of *KmTPX1*. Overnight cultures of two strains were inoculated into 100 mL SC-His medium supplemented with 0.2 mM or 3 mM H_2_O_2_, adjusting the initial cell density to around 0.05 g/L of DCW. The fermentation was performed at 30 °C and 150 rpm, and the samples were taken every 12 h to test cell growth.

The potential of an enhanced acetic acid tolerance was tested by serial dilution assay as was described above with the concentration of acetic acid increasing from 1.5 to 5 g/L. Effects of pH on acetic acid tolerance were evaluated by adjusting the initial pH to 4.5 with 3 N NaOH. The concentrations of acetic acid in SD-His plates were 1.5, 4, 5, and 10 g/L, respectively (Fig. 4).

### Laboratory-scale batch fermentation

To evaluate the fermentative performance of strain TPX1, batch fermentation was conducted in bioreactors for 60 h. Pre-cultures of yeast cells were carried out in 100 mL of SC-His medium in 250-mL flasks for 16–18 h, and then 5% of cells were inoculated in a 3-L fermenter with 1 L of SC-His medium containing 50 g/L of glucose and FAF mixture (0.3 g/L formic acid, 1.2 g/L acetic acid, and 0.5 g/L furfural) at 30 °C and 150 rpm. The initial cell density was adjusted to 0.08–0.09 g/L of DCW, and the aeration rate was kept at 0.05 vvm.

To test the effect of initial pH on the fermentation and tolerance of TPX1 strain, batch fermentation was performed at an elevated concentration of acetic acid in FAF inhibitors (0.3 g/L formic acid, 5 g/L acetic acid, and 0.5 g/L furfural). Before inoculation, the initial pH of the medium with inhibitors was adjusted to around 4.5, and other environmental parameters were kept the same as above.

Samples were taken every 12 h to test cell growth, sugar and ethanol.

### Bioinformatics analysis

Expression pattern analysis of differentially expressed genes related to oxidative stress from transcriptome of *K. marxianus* Y179 [20] was clustered using Cluster software [39] and Java TreeView software [40]. The hierarchical clustering of the chosen experimental conditions and genes was carried out using Euclidean Distance as the formula of the distance matrix. Evolutionary tree of some typical 2-Cys Prxs from Y179 and its allied species using *MEGA 4* software [41], and amino acid sequences applied are listed as follows: *KmTSA1/KmTPX1*, Tsa1 protein from *K. marxianus* Y179 in this study; *KlTSA1*, Tsa1 protein from *K. lactis* (Accession No. XP_451603.1); *ScTSA1*, Tsa1 protein from *S. cerevisiae* (Accession No. NP_013684.1); *ScTSA2*, Tsa2 protein from *S. cerevisiae* (Accession No. NP_010741.1); *SsTSA1*, Tsa1 protein from *S. stipitis* (Accession No. XP_001382622.1); *CtTSA1*, Tsa1 protein from *C. tropicalis* (Accession No. XP_002547929.1); *ScPRX1*, Pxr1 protein from *S. cerevisiae* (Accession No. NP_009489.1); *ScAHP1*, Ahp1 protein from *S. cerevisiae* (Accession No. NP_013210.1); and *ScDOT5*, Dot5 protein from *S. cerevisiae* (Accession No. NP_012255.3). Alignment was conducted using the National Center for Biotechnology Information (NCBI) Basic Local Alignment Search Tool (BLAST) software (http://clustalw.ddbj.nig.ac.jp/).

### ROS level analysis

Levels of ROS inside cells were tested by a common method using 2′,7′-dichlorofluorescein diacetate (DCFH-DA, Sigma-35845, dissolved in absolute ethanol) as an indicator. Cells after pre-culture were cultivated for 16–18 h in SD-His medium with FAF (0.3 g/L formic acid, 1.2 g/L acetic acid, and 0.5 g/L furfural) at 30 °C and 150 rpm. Cell pellets were washed twice with distilled water and then re-suspended in 0.5 mL of 10 mM PBS (pH 7.0) containing 10 μM DCFH-DA. After incubation at 37 °C for 60 min, fluorescence was measured by a Multiskan spectrum microplate spectrophotometer (PerkinElmer, USA).

### Analytical methods

The cell concentration was measured in dry cell weight (DCW) following the previous protocol [42]. Concentrations of glucose, ethanol, and glycerol were analyzed by Aminex HPX-87H column (300 × 7.8 mm; Bio-Rad, Hercules) in HPLC system, which used 0.01 mol/L H_2_SO_4_ as the mobile phase and was eluted at 50 °C with a flow rate of 0.5 mL/min. All analyses in this study were done in duplicate except indicated, and the mean values are shown in the Results and discussion section.

## Additional files

**Additional file 1: Figure S1.** Construction of overexpressing vector and subsequent verification. a) The schematic of overexpressing vector containing KmTPX1 gene and its own promoter. b) PCR and restriction enzyme digestion verification with a band of 1042 bp. c) Relative abundance of KmTPX1 overexpression in SC-His medium by real-time quantitative PCR technology.

**Additional file 2: Figure S2.** Fermentation profile in *KmTPX1*-expressing *S. cerevisiae* cells during batch ethanol production process under FAF stress in flasks. a) Growth behavior of two strains at first 60 h. b) Glucose consumption and ethanol production under FAF stress. Cells were pre-cultured in SC-His medium containing 1 mM H_2_O_2_ for 16–18 h. 1% of seed culture was inoculated into a 250 mL flask with a 100 mL working volume. Data are given as means ± SD, n = 2.

**Additional file 3: Table S1.** Fermentative performance under FAF inhibitors within 72 h in flasks.

## Abbreviations

ROS: reactive oxygen species
DEGs: differentially expressed genes
Prxs: peroxiredoxins
ORP: oxidoreduction potential (mV)
FAF: a mixture of formic acid, acetic acid and furfural inhibitors
FAFH: a mixture of formic acid, acetic acid, furfural, and 5-HMF inhibitors
SC: synthetic complete medium
SC-His: SC medium without histidine
DCW: dry cell weight (g/L)
DCFH-DA: 2′,7′-dichlorofluorescein diacetate

## References

[1] Palmqvist E, Hahn-Hägerdal B (2000). Fermentation of lignocellulosic hydrolysates. II: inhibitors and mechanisms of inhibition. Bioresour Technol.

[2] Lee HJ, Lim WS, Lee JW (2013). Improvement of ethanol fermentation from lignocellulosic hydrolysates by the removal of inhibitors. J Ind Eng Chem.

[3] Chen R, Dou J (2016). Biofuels and bio-based chemicals from lignocellulose: metabolic engineering strategies in strain development. Biotechnol Lett.

[4] Giannattasio S, Guaragnella N, Ždralević M, Marra E (2013). Molecular mechanisms of *Saccharomyces cerevisiae* stress adaptation and programmed cell death in response to acetic acid. Front Microbiol.

[5] Kim SK, Jin YS, Choi IG, Park YC, Seo JH (2015). Enhanced tolerance of *Saccharomyces cerevisiae* to multiple lignocellulose-derived inhibitors through modulation of spermidine contents. Metab Eng.

[6] Ibraheem O, Ndimba BK (2013). Molecular adaptation mechanisms employed by ethanologenic bacteria in response to lignocellulose-derived inhibitory compounds. Int J Biol Sci.

[7] Roe AJ, McLaggan D, Davidson I, O’Byrne C, Booth IR (1998). Perturbation of anion balance during inhibition of growth of *Escherichia coli* by weak acids. J Bacteriol.

[8] Kumar V, Hart AJ, Keerthiraju ER, Waldron PR, Tucker GA, Greetham D (2015). Expression of mitochondrial cytochrome C oxidase chaperone gene (*COX20*) improves tolerance to weak acid and oxidative stress during yeast fermentation. PLoS ONE.

[9] Wang X, Bai X, Chen DF, Chen FZ, Li BZ, Yuan YJ (2015). Increasing proline and myo-inositol improves tolerance of *Saccharomyces cerevisiae* to the mixture of multiple lignocellulose-derived inhibitors. Biotechnol Biofuels.

[10] Allen SA, Clark W, McCaffery JM, Cai Z, Lanctot A, Slininger PJ (2010). Furfural induces reactive oxygen species accumulation and cellular damage in *Saccharomyces cerevisiae*. Biotechnol Biofuels.

[11] Almeida JR, Röder A, Modig T, Laadan B, Lidén G, Gorwa-Grauslund MF (2008). NADH-vs NADPH-coupled reduction of 5-hydroxymethyl furfural (HMF) and its implications on product distribution in *Saccharomyces cerevisiae*. Appl Microbiol Biotechnol.

[12] Petersson A, Almeida JR, Modig T, Karhumaa K, Hahn-Hägerdal B, Gorwa-Grauslund MF (2006). A 5-hydroxymethyl furfural reducing enzyme encoded by the Saccharomyces cerevisiae ADH6 gene conveys HMF tolerance. Yeast.

[13] Moon J, Liu ZL (2012). Engineered NADH-dependent GRE2 from *Saccharomyces cerevisiae* by directed enzyme evolution enhances HMF reduction using additional cofactor NADPH. Enzyme Microb Technol.

[14] Liu ZL, Moon J (2009). A novel NADPH-dependent aldehyde reductase gene from *Saccharomyces cerevisiae* NRRL Y-12632 involved in the detoxification of aldehyde inhibitors derived from lignocellulosic biomass conversion. Gene.

[15] Almeida JR, Modig T, Petersson A, Hähn-Hägerdal B, Lidén G, Gorwa-Grauslund MF (2007). Increased tolerance and conversion of inhibitors in lignocellulosic hydrolysates by *Saccharomyces cerevisiae*. J Chem Technol Biotechnol.

[16] Yi X, Gu H, Gao Q, Liu ZL, Bao J (2015). Transcriptome analysis of *Zymomonas mobilis* ZM4 reveals mechanisms of tolerance and detoxification of phenolic aldehyde inhibitors from lignocellulose pretreatment. Biotechnol Biofuels.

[17] Ma M, Liu ZL (2010). Comparative transcriptome profiling analyses during the lag phase uncover *YAP1*, *PDR1*, *PDR3*, *RPN4*, and *HSF1* as key regulatory genes in genomic adaptation to the lignocellulose derived inhibitor HMF for *Saccharomyces cerevisiae*. BMC Genom.

[18] Zhou Q, Liu ZL, Ning K, Wang A, Zeng X, Xu J (2014). Genomic and transcriptome analyses reveal that MAPK-and phosphatidylinositol-signaling pathways mediate tolerance to 5-hydroxymethyl-2-furaldehyde for industrial yeast *Saccharomyces cerevisiae*. Sci Rep.

[19] Yang J, Ding MZ, Li BZ, Liu ZL, Wang X, Yuan YJ (2012). Integrated phospholipidomics and transcriptomics analysis of *Saccharomyces cerevisiae* with enhanced tolerance to a mixture of acetic acid, furfural, and phenol. OMICS.

[20] Gao JQ, Yuan WJ, Li YM, Xiang RJ, Hou SB, Zhong SJ (2015). Transcriptional analysis of *Kluyveromyces marxianus* for ethanol production from inulin using consolidated bioprocessing technology. Biotechnol Biofuels.

[21] Toledano MB, Huang B (2016). Microbial 2-Cys peroxiredoxins: insights into their complex physiological roles. Mol Cells.

[22] Tachibana T, Okazaki S, Murayama A, Naganuma A, Nomoto A, Kuge S (2009). A major peroxiredoxin-induced activation of Yap1 transcription factor is mediated by reduction-sensitive disulfide bonds and reveals a low level of transcriptional activation. J Biol Chem.

[23] Rhee SG (2016). Overview on Peroxiredoxin. Mol Cells.

[24] Iraqui I, Kienda G, Soeur J, Faye G, Baldacci G, Kolodner RD, Huang ME (2009). Peroxiredoxin Tsa1 is the key peroxidase suppressing genome instability and protecting against cell death in *Saccharomyces cerevisiae*. PLoS Genet.

[25] Cui J, Lee SY, Jang HH (2015). Yeast 2-Cys peroxiredoxin Tsa1 protects cells from DNA damage-induced reactive oxygen species through peroxidase activity. J Korean Soc Appl Biol.

[26] Perkins A, Nelson KJ, Parsonage D, Poole LB, Karplus PA (2015). Peroxiredoxins: guardians against oxidative stress and modulators of peroxide signaling. Trends Biochem Sci.

[27] Tairum CA, de Oliveira MA, Horta BB, Zara FJ, Netto LE (2012). Disulfide biochemistry in 2-cys peroxiredoxin: requirement of Glu50 and Arg146 for the reduction of yeast Tsa1 by thioredoxin. J Mol Biol.

[28] Wong CM, Siu KL, Jin DY (2004). Peroxiredoxin-null yeast cells are hypersensitive to oxidative stress and are genomically unstable. J Biol Chem.

[29] Kim IS, Kim YS, Yoon HS (2013). Expression of salt-induced 2-Cys peroxiredoxin from *Oryza sativa* increases stress tolerance and fermentation capacity in genetically engineered yeast *Saccharomyces cerevisiae*. Appl Microbiol Biotechnol.

[30] Li H, Wei JC (2016). Functional analysis of thioredoxin from the desert lichen-forming fungus, *Endocarpon pusillum* Hedwig, reveals its role in stress tolerance. Sci Rep..

[31] Zhang M, Shi J, Jiang L (2015). Modulation of mitochondrial membrane integrity and ROS formation by high temperature in *Saccharomyces cerevisiae*. Electron J Biotechnol.

[32] Martinez A, Rodriguez ME, Wells ML, York SW, Preston JF, Ingram LO (2001). Detoxification of dilute acid hydrolysates of lignocellulose with lime. Biotechnol Progr..

[33] Mussatto SI, Roberto IC (2004). Alternatives for detoxification of diluted-acid lignocellulosic hydrolyzates for use in fermentative processes: a review. Bioresour Technol.

[34] Zhang MM, Zhao XQ, Cheng C, Bai FW (2015). Improved growth and ethanol fermentation of *Saccharomyces cerevisiae* in the presence of acetic acid by overexpression of *SET5* and *PPR1*. Biotechnol J.

[35] Sikorski RS, Hieter P (1989). A system of shuttle vectors and yeast host strains designed for efficient manipulation of DNA in *Saccharomyces cerevisiae*. Genetics.

[36] Green MR, Sambrook J (2012). Molecular cloning: a laboratory manual (Vol 1).

[37] Cohen SN, Chang AC, Hsu L (1972). Nonchromosomal antibiotic resistance in bacteria: genetic transformation of *Escherichia coli* by R-factor DNA. P Natl Acad Sci.

[38] Gietz RD, Schiestl RH (2007). High-efficiency yeast transformation using the LiAc/SS carrier DNA/PEG method. Nat Protoc.

[39] de Hoon MJL, Imoto S, Nolan J, Miyano S (2004). Open source clustering software. Bioinformatics.

[40] Saldanha J (2004). Java Treeview-extensible visualization of microarray data. Bioinformatics.

[41] Tamura K, Dudley J, Nei M, Kumar S (2007). MEGA4: molecular evolutionary genetics analysis (MEGA) software version 4.0. Mol Biol Evol.

[42] Bai FW, Chen LJ, Anderson WA, Moo-Young M (2004). Parameter oscillations in a very high gravity medium continuous ethanol fermentation and their attenuation on a multistage packed column bioreactor system. Biotechnol Bioeng.

