# Supplementary figures and images for: Enhanced fermentative performance under stresses of multiple lignocellulose-derived inhibitors by overexpression of a typical 2-Cys peroxiredoxin from Kluyveromyces marxianus
Source: Biotechnol Biofuels. 2017 Mar 28;10:79. doi: 10.1186/s13068-017-0766-4 (PMC5370469; doi:10.1186/s13068-017-0766-4)

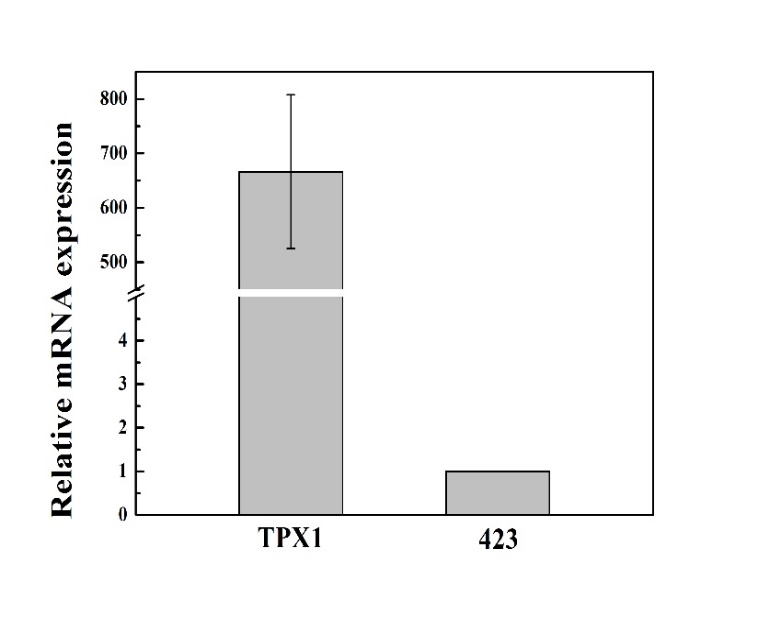


**c**

***Sac*I**

***Sac*II**

***pKmTPX1p***

***KmTPX1***


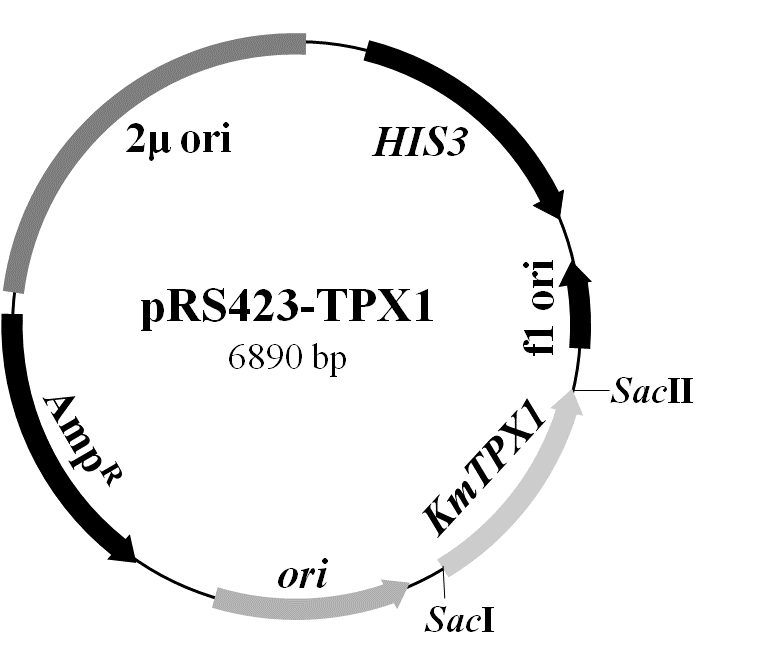

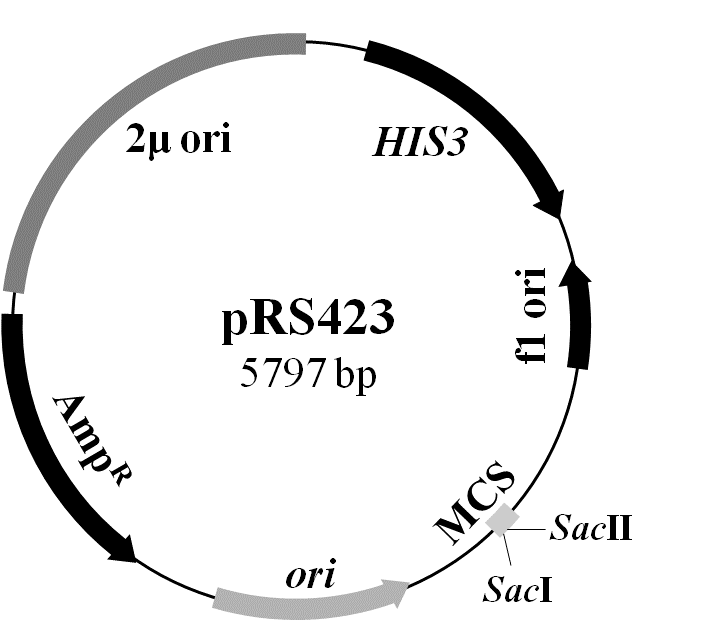

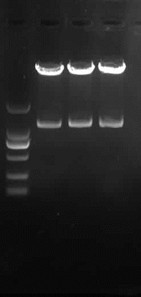


**1042 bp**

**1042 bp**


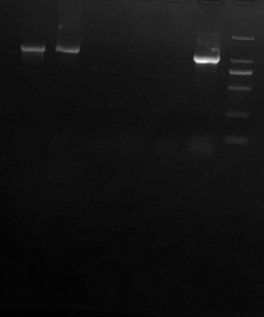


**a**

**b**

**Figure S1**

Supplement: Supplementary file 1 — Additional file 1: Figure S1. Construction of overexpressing vector and subsequent verification. a) The schematic of overexpressing vector containing KmTPX1 gene and its own promoter. b) PCR and restriction enzyme digestion verification with a band of 1042 bp. c) Relative abundance of KmTPX1 overexpression in SC-His medium by real-time quantitative PCR technology. [file 13068_2017_766_MOESM1_ESM.doc]

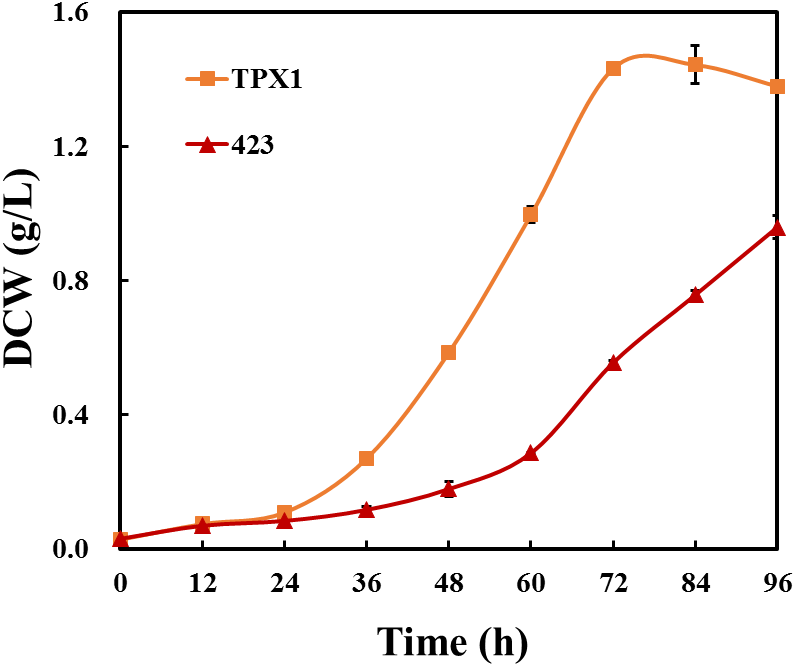


**a**

**b**

**Figure S2**

Supplement: Supplementary file 2 — Additional file 2: Figure S2. Fermentation profile in KmTPX1-expressing S. cerevisiae cells during batch ethanol production process under FAF stress in flasks. a) Growth behavior of two strains at first 60 h. b) Glucose consumption and ethanol production under FAF stress. Cells were pre-cultured in SC-His medium containing 1 mM H2O2 for 16–18 h. 1% of seed culture was inoculated into a 250 mL flask with a 100 mL working volume. Data are given as means ± SD, n = 2. [file 13068_2017_766_MOESM2_ESM.doc]
