# Supplementary material for: Enhanced fermentative performance under stresses of multiple lignocellulose-derived inhibitors by overexpression of a typical 2-Cys peroxiredoxin from Kluyveromyces marxianus
Source: Biotechnol Biofuels. 2017 Mar 28;10:79. doi: 10.1186/s13068-017-0766-4 (PMC5370469; doi:10.1186/s13068-017-0766-4)
Supplement: Supplementary file 3 — Additional file 3: Table S1. Fermentative performance under FAF inhibitors within 72 h in flasks. [file 13068_2017_766_MOESM3_ESM.doc]

Table S1 Fermentative performance under FAF inhibitors within 72 h in flasks.

|  | TPX1 | 423 |
| --- | --- | --- |
| Lag phase, h | 24 | 48 |
| Total glucose, g/L | 50.67±0.09 | 51.71±1.32 |
| Residual glucose, g/L | 1.64±1.59 | 37.09±2.43 |
| ^a^Glucose consumption rate, g/L/h | 0.99±0.03 | 0.28±0.05 |
| Glycerol, g/L | 1.94±0.03 | 0.77±0.05 |
| Ethanol, g/L | 21.99±0.76 | 7.28±0.31 |
| ^a^Etahnol generation rate, g/L/h | 0.43±0.02 | 0.13±0.01 |
| Productivity, g/L/h | 0.31±0.01 | 0.10±0.00 |

^a^Data was calculated between 24 and 72 h
